# Supplementary material for: Sex Disparities in In‐Hospital Outcomes After Percutaneous Coronary Intervention (PCI) in Patients With Acute Myocardial Infarction and a History of Coronary Artery Bypass Grafting (CABG): A Cross‐Sectional Study
Source: Health Sci Rep. 2024 Dec 19;7(12):e70292. doi: 10.1002/hsr2.70292 (PMC11659193; doi:10.1002/hsr2.70292)
Supplement: Supplementary file 1 — Supporting information. [file HSR2-7-e70292-s001.docx]

**Supplementary Material**

**Table S1: STROBE Statement—checklist of items that should be included in reports of observational studies**

|  | **Item No** | **Recommendation** |
| --- | --- | --- |
| **Title and abstract** | **1** | **(*a*) Indicate the study’s design with a commonly used term in the title or the abstract** |
|  |  | **(*b*) Provide in the abstract an informative and balanced summary of what was done and what was found** |
| **Introduction** | | |
| **Background/****rationale** | **2** | **Explain the scientific background and rationale for the investigation being reported** |
| **Objectives** | **3** | **State specific objectives, including any prespecified hypotheses** |
| **Methods** | | |
| **Study design** | **4** | **Present key elements of study design early in the paper** |
| **Setting** | **5** | **Describe the setting, locations, and relevant dates, including periods of recruitment, exposure, follow-up, and data collection** |
| **Participants** | **6** | **(*a*) *Cohort study*—Give the eligibility criteria, and the sources and methods of selection of participants. Describe methods of follow-up**  ***Case-control study*—Give the eligibility criteria, and the sources and methods of case ascertainment and control selection. Give the rationale for the choice of cases and controls**  ***Cross-sectional study*—Give the eligibility criteria, and the sources and methods of selection of participants** |
|  |  | **(*b*) *Cohort study*—For matched studies, give matching criteria and number of exposed and unexposed**  ***Case-control study*—For matched studies, give matching criteria and the number of controls per case** |
| **Variables** | **7** | **Clearly define all outcomes, exposures, predictors, potential confounders, and effect modifiers. Give diagnostic criteria, if applicable** |
| **Data sources/** **measurement** | **8******* | **For each variable of interest, give sources of data and details of methods of assessment (measurement). Describe comparability of assessment methods if there is more than one group** |
| **Bias** | **9** | **Describe any efforts to address potential sources of bias** |
| **Study size** | **10** | **Explain how the study size was arrived at** |
| **Quantitative** **variables** | **11** | **Explain how quantitative variables were handled in the analyses. If applicable, describe which groupings were chosen and why** |
| **Statistical** **methods** | **12** | **(*a*) Describe all statistical methods, including those used to control for confounding** |
|  |  | **(*b*) Describe any methods used to examine subgroups and interactions** |
|  |  | **(*c*) Explain how missing data were addressed** |
|  |  | **(*d*) *Cohort study*—If applicable, explain how loss to follow-up was addressed**  ***Case-control study*—If applicable, explain how matching of cases and controls was addressed**  ***Cross-sectional study*—If applicable, describe analytical methods taking account of sampling strategy** |
|  |  | **(*e*) Describe any sensitivity analyses** |
| **Results** |  |  |
| **Participants** | **13*** | **(a) Report numbers of individuals at each stage of study—eg numbers potentially eligible, examined for eligibility, confirmed eligible, included in the study, completing follow-up, and analysed** |
|  |  | **(b) Give reasons for non-participation at each stage** |
|  |  | **(c) Consider use of a flow diagram** |
| **Descriptive data** | **14*** | **(a) Give characteristics of study participants (eg demographic, clinical, social) and information on exposures and potential confounders** |
|  |  | **(b) Indicate number of participants with missing data for each variable of interest** |
|  |  | **(c) *Cohort study*—Summarise follow-up time (eg, average and total amount)** |
| **Outcome data** | **15*** | ***Cohort study*—Report numbers of outcome events or summary measures over time** |
|  |  | ***Case-control study—*Report numbers in each exposure category, or summary measures of exposure** |
|  |  | ***Cross-sectional study—*Report numbers of outcome events or summary measures** |
| **Main results** | **16** | **(*a*) Give unadjusted estimates and, if applicable, confounder-adjusted estimates and their precision (eg, 95% confidence interval). Make clear which confounders were adjusted for and why they were included** |
|  |  | **(*b*) Report category boundaries when continuous variables were categorized** |
|  |  | **(*c*) If relevant, consider translating estimates of relative risk into absolute risk for a meaningful time period** |
| **Other analyses** | **17** | **Report other analyses done—eg analyses of subgroups and interactions, and sensitivity analyses** |
| **Discussion** |  |  |
| **Key results** | **18** | **Summarise key results with reference to study objectives** |
| **Limitations** | **19** | **Discuss limitations of the study, taking into account sources of potential bias or imprecision. Discuss both direction and magnitude of any potential bias** |
| **Interpretation** | **20** | **Give a cautious overall interpretation of results considering objectives, limitations, multiplicity of analyses, results from similar studies, and other relevant evidence** |
| **Generalisability** | **21** | **Discuss the generalisability (external validity) of the study results** |
| **Other information** |  |  |
| **Funding** | **22** | **Give the source of funding and the role of the funders for the present study and, if applicable, for the original study on which the present article is based** |

***Give information separately for cases and controls in case-control studies and, if applicable, for exposed and unexposed groups in cohort and cross-sectional studies.**

**Note: An Explanation and Elaboration article discusses each checklist item and gives methodological background and published examples of transparent reporting. The STROBE checklist is best used in conjunction with this article (freely available on the Web sites of PLoS Medicine at http://www.plosmedicine.org/, Annals of Internal Medicine at http://www.annals.org/, and Epidemiology at http://www.epidem.com/). Information on the STROBE Initiative is available at** [**www.strobe-statement.org**](http://www.strobe-statement.org)**.**

**Table S2: International Classification of Diseases, 10th Revision, Clinical Modification/Procedure Coding System (ICD-10 CM/PCS) Codes**

| **Variables** | **ICD-10 CM and PCS codes** |
| --- | --- |
| **AMI** | I21.x |
| **Prior CABG** | Z95.1, I257xx, I25810, I25812 |
| **Prior PCI** | Z9861, Z955 |
| **CABG** | 0210, 0211, 0212, 0213 |
| **PCI** | 0270, 0271, 0272, 0273 |
| **Known CAD** | I25118, I25119 |
| **Family history of CAD** | Z82.49 |
| **Prior MI** | I252, I22xx |
| **Prior CVA** | Z86.73 |
| **Prior PPM or ICD** | Z95.0, Z95.810 |
| **Smoking** | F17xZ72.0, Z87891 |
| **Dyslipidemia** | E78x |
| **Carotid artery disease** | I65.2X |
| **Atrial fibrillation** | I480, I481, I4811, I4819, I4820, I4821, I4891 |
| **Ventricular fibrillation** | I49.01 |
| **Congestive Heart Failure** | I50x, I110, I130, I132, I255, I43, I420, I425, I426, I427, I428, I429, P290, I099 |
| **Alcohol abuse** | G621, I426, K292, K700, K703, K709, Z7141, F10x, E52 |
| **Deficiency anemia** | D508, D509, D51, D52x, D53x |
| **Chronic blood loss anemia** | D500 |
| **Rheumatoid arthritis/ collagen vascular diseases** | M05, M06, M08, M30, M32, M33, M34, M35, M45, L940, L941, L943, M120, M123, M310, M311, M312, M313, M461, M468, M469 |
| **Coagulopathy** | D65, D66, D67, D68, D691, D693, D694, D695, D696 |
| **Depression** | F32, F33, F313, F314, F315, F341, F432 |
| **Diabetes mellitus** | E100, E101, E109, E110, E111, E119, E120, E121, E129, E130, E131, E139, E140, E141, E149, E102, E103, E104, E105, E106, E107, E108, E112, E113, E114, E115, E116, E117, E118, E122, E123, E124, E125, E126, E127, E128, E132, E133, E134, E135, E136, E137, E138, E142, E143, E144, E145, E146, E147, E148 |
| **Hypertension** | I10, I11, I12, I13, I15 |
| **Drug abuse** | F11, F12, F13, F14, F15, F16, F18, F19, Z7151 |
| **Hypothyroidism** | E02, E03, E890, E01, E01 |
| **Chronic pulmonary disease** | I272, I278, I279, J684, J701, J703, J40, J41, J42, J43, J44, J45, J46, J47, J60, J61, J62, J63, J64, J65, J66, J67 |
| **Pulmonary circulation disorders** | I26, I27, I280, I288, I289 |
| **Coagulopathy** | D65, D66, D67, D68, D691, D693, D694, D695, D696 |
| **Liver disease** | I864, I982, K711, K713, K714, K715, K717, K760, K762, K763, K764, K765, K766, K767, K768,  K769, Z944, K70, K72, K73, K74, B18, I85 |
| **Fluid and electrolyte disorder** | E86, E87, E222 |
| **Other neurological disorders** | G254, G255, G312, G318, G319, G931, G934,  R470, G10, G11, G12, G13, G20, G21, G22, G32, G35, G36, G37, G40, G41, R56 |
| **Peripheral vascular disease** | I70, I71, I731, I738, I739, I771, I790, I792,  K551, K558, K559, Z958, Z959 |
| **Valvular disease** | I70, I71, I731, I738, I739, I771, I790, I792, K551, K558, K559, Z958, Z959 |
| **Chronic renal failure** | I120, I131, N250, Z490, Z491, Z492, Z940, Z992, N18, N19 |
| **Acute renal failure** | N17.x |
| **Cancer** | C00, C01, C02, C03, C04, C05, C06, C07, C08, C09, C10, C11, C12, C13, C14, C15, C16, C17, C18, C19, C20, C21, C22, C23, C24, C25, C26, C30, C31, C32, C33, C34, C37, C38, C39, C40, C41, C43, C45, C46, C47, C48, C49, C50, C51, C52, C53, C54, C55, C56, C57, C58, C60, C61, C62, C63, C64, C65, C66, C67, C68, C69, C70, C71, C72, C73, C74, C75, C76, C97, C77, C78, C79, C80 |
| **Lymphoma** | C81, C82, C83, C84, C85, C86, C88, C90 |
| **Paralysis** | G041, G114, G801, G802, G830, G831, G832, G833, G834, G839, G81, G82 |
| **Peptic ulcer** | K257, K259, K267, K269, K277, K279, K287, K289 |
| **Weight loss** | E40, E41, E42, E43, E44, E45, E46, R64, R634 |
| **Cardiac arrhythmias** | I40, I41，I42, I43, I45.x, R000, R001, R002, R008, T821, Z450, Z950, I47.X, I480, I481, I4811, I4819, I4820, I4821, I4891 I48.3, I48.4, I48.92, I49.x, I47.2X |
| **Obesity** | E66.0, E66.1, E66.2, E66.8, E66.9, Z68.3, Z68.4 |
| **Ischemic stroke** | I63.xx, |
| **Hemorrhagic stroke** | I60.x, I61.x, I62.x |
| **Intracardiac thrombus** | I51.3, I23.6 |
| **Cardiogenic shock** | R57.0 |
| **Cardiac arrest** | I46, I97. 12, I97.71 |
| **Vasopressor use** | 3E030XZ, 3E033XZ, 3E040XZ, 3E043XZ, 3E050XZ, 3E053XZ, 3E060XZ, 3E063XZ |
| **Coronary angiography** | 4A023N7 |
| **Invasive hemodynamic monitoring** | 4A1239Z, 4A0239Z, 4A023N6, 4A023N8, 4A033J3, 4A033B3, 4A03353, 4A13353, 4A133B3, 4A133J3, 02HP32Z |
| **MCS** | IABP: 5A02210, 5A02110  Impella: 5A0221D，5A0211D  ECMO: 5A1522F, 5A1522G, 5A15A2F, 5A15A2G, 5A15223 |
| **Hemopericardium** | I31.2 |
| **Pericardiocentesis** | 0W9D30Z, 0W9D3ZX, 0W9D3ZZ, 0W9D40Z, 0W9D4ZX, 0W9D4ZZ, 0W9D0ZX, 0W9D0ZZ |
| **Cardiac tamponade** | I31.4 |
| **Gastrointestinal Bleeding** | K92.0-92.2, K25.0-25.2, K25.4-25.6, K26.0-26.2, K27.0-27.2, K27.4-27.6, K28.0-28.2, K28.4-28.6 |
| **Blood transfusion** | 30230H*, 30233H*, 30233k*, 30230k*, 30233L*, 30230L*, 30233M*, 30230M*, 30233N*, 30230N*, 30233P*, 30230P*, 30233R*,30230R*, 30233T*, 30230T*, 30233V*, 30230V*, 30233W*, 30230W* |

**Table S3: 31 AHRQ Elixhauser comorbidity measures**

|  | **Variables** |
| --- | --- |
| 1 | Congestive Heart Failure |
| 2 | AIDS |
| 3 | Alcohol abuse |
| 4 | Deficiency anemia |
| 5 | Rheumatoid arthritis/ collagen vascular diseases |
| 6 | Chronic blood loss anemia |
| 7 | Chronic pulmonary disease |
| 8 | Coagulopathy |
| 9 | Depression |
| 10 | Diabetes mellitus uncomplicated |
| 11 | Diabetes mellitus complicated |
| 12 | Hypertension uncomplicated |
| 13 | Hypertension complicated |
| 14 | Drug abuse |
| 15 | Hypothyroidism |
| 16 | Liver disease |
| 17 | Lymphoma |
| 18 | Fluid and electrolyte disorder |
| 19 | Metastatic cancer |
| 20 | Solid tumor without metastasis |
| 21 | Other neurological disorders |
| 22 | Paralysis |
| 23 | Peripheral vascular disease |
| 24 | Psychoses |
| 25 | Pulmonary circulation disorders |
| 26 | Chronic renal failure |
| 27 | Peptic ulcer |
| 28 | Valvular disease |
| 29 | Weight loss |
| 30 | Obesity |
| 31 | Cardiac arrhythmias |

**Table S4: Matching variables in propensity score analysis**

| Y | X | **Matching variables** |
| --- | --- | --- |
| In-hospital mortality, MACCEs, Bleeding, Stroke | Sex | Age**,** race**,** primary pay**,** ZIP income, hospital region**,** teaching status of the hospital, bed-size of the hospital, smoking, dyslipidemia, family history of coronary heart disease, prior mi, atrial fibrillation, congestive heart failure, deficiency anemia, chronic blood loss anemia, chronic pulmonary disease, coagulopathy, depression, diabetes mellitus，hypertension, hypothyroidism, liver disease, fluid and electrolyte disorder, other neurological disorders, pulmonary circulation disorder, chronic renal failure, valvular disease, obesity, cardiogenic shock, ventricular fibrillation, cardiac arrest, acute renal failure, coronary angiography, invasive hemodynamic monitoring, vasopressor use, mechanical circulatory support |

**Table S5: Baseline characteristics of female versus male patients in the cohort after propensity score matching**

| Variables | Male (n=3700) | Female (n=3700) | SMD |
| --- | --- | --- | --- |
| Age, years | 70.02 ± 10.41 | 70.38 ± 11.12 | 0.03 |
| Race |  |  | 0.26 |
| White | 3024 (81.73) | 2768 (74.81) |  |
| Black | 205 (5.54) | 471 (12.73) |  |
| Hispanic | 269 (7.27) | 287 (7.76) |  |
| Other | 202 (5.46) | 174 (4.70) |  |
| Primary payer |  |  | 0.23 |
| Medicare/ Medicaid | 2755 (74.46) | 3049 (82.41) |  |
| Private insurance | 688 (18.59) | 495 (13.38) |  |
| Self-pay | 91 (2.46) | 96 (2.59) |  |
| No charge/Other | 166 (4.49) | 60 (1.62) |  |
| ZIP income |  |  | 0.10 |
| 0-25th | 1196 (32.32) | 1349 (36.46) |  |
| 26-50th | 1104 (29.84) | 1067 (28.84) |  |
| 51-75th | 822 (22.22) | 788 (21.30) |  |
| 76-100th | 578 (15.62) | 496 (13.41) |  |
| Hospital region |  |  | 0.05 |
| Northeast | 561 (15.16) | 559 (15.11) |  |
| Midwest | 918 (24.81) | 930 (25.14) |  |
| South | 1807 (48.84) | 1855 (50.14) |  |
| West | 414 (11.19) | 356 (9.62) |  |
| Teaching status of the hospital |  |  | 0.05 |
| Rural hospital | 250 (6.76) | 261 (7.05) |  |
| Urban non-teaching | 823 (22.24) | 753 (20.35) |  |
| Urban teaching | 2627 (71.00) | 2686 (72.59) |  |
| Bed-size of the hospital |  |  | 0.00 |
| Small | 568 (15.35) | 571 (15.43) |  |
| Medium | 1116 (30.16) | 1112 (30.05) |  |
| Large | 2016 (54.49) | 2017 (54.51) |  |
| Comorbidities |  |  |  |
| Known CAD | 368 (9.95) | 312 (8.43) | 0.05 |
| Family history of CAD | 471 (12.73) | 448 (12.11) | 0.02 |
| Prior MI | 1045 (28.24) | 943 (25.49) | 0.06 |
| Prior CVA | 393 (10.62) | 452 (12.22) | 0.05 |
| Prior PPM or ICD | 185 (5.00) | 170 (4.59) | 0.02 |
| Smoking | 1850 (50.00) | 1564 (42.27) | 0.16 |
| Obesity | 677 (18.30) | 749 (20.24) | 0.05 |
| Alcohol abuse | 89 (2.41) | 14 (0.38) | 0.17 |
| Drug abuse | 72 (1.95) | 59 (1.59) | 0.03 |
| Dyslipidemia | 3069 (82.95) | 2999 (81.05) | 0.05 |
| Carotid artery disease | 131 (3.54) | 135 (3.65) | 0.01 |
| Atrial fibrillation | 692 (18.70) | 672 (18.16) | 0.01 |
| Ventricular fibrillation | 91 (2.46) | 71 (1.92) | 0.04 |
| Congestive heart failure | 1304 (35.24) | 1195 (32.30) | 0.06 |
| Deficiency anemia | 112 (3.03) | 150 (4.05) | 0.06 |
| Chronic blood loss anemia | 17 (0.46) | 24 (0.65) | 0.03 |
| Coagulopathy | 225 (6.08) | 155 (4.19) | 0.09 |
| Hypertension | 2195 (59.32) | 2098 (56.70) | 0.05 |
| Diabetes | 1971 (53.27) | 2207 (59.65) | 0.13 |
| Depression | 379 (10.24) | 521 (14.08) | 0.12 |
| Chronic pulmonary disease | 958 (25.89) | 1133 (30.62) | 0.11 |
| Pulmonary circulation disorders | 11 (0.30) | 11 (0.30) | 0.00 |
| Intracardiac thrombus | 15 (0.41) | 5 (0.14) | 0.05 |
| Hypothyroidism | 526 (14.22) | 774 (20.92) | 0.18 |
| Liver disease | 107 (2.89) | 105 (2.84) | 0.00 |
| Rheumatoid arthritis/collagen vascular diseases | 63 (1.70) | 157 (4.24) | 0.15 |
| Cancer | 64 (1.73) | 48 (1.30) | 0.04 |
| Fluid and electrolyte disorders | 772 (20.86) | 909 (24.57) | 0.09 |
| Other neurological disorders | 218 (5.89) | 236 (6.38) | 0.02 |
| Peripheral vascular disease | 747 (20.19) | 730 (19.73) | 0.01 |
| Chronic kidney disease | 1169 (31.59) | 1203 (32.51) | 0.02 |
| Acute renal failure | 778 (21.03) | 795 (21.49) | 0.01 |
| Valvular disease | 649 (17.54) | 784 (21.19) | 0.09 |
| Cardiogenic shock | 203 (5.49) | 207 (5.59) | 0.84 |
| Cardiac arrest | 91 (2.46) | 94 (2.54) | 0.00 |
| Elixhauser comorbidities |  |  | 0.09 |
| 0 | 70 (1.89) | 44 (1.19) |  |
| 1-4 | 2536 (68.54) | 2435 (65.81) |  |
| >4 | 1094 (29.57) | 1221 (33.00) |  |
| Vasopressor use | 28 (0.76) | 23 (0.62) | 0.02 |
| Coronary angiography | 2901 (78.41) | 2924 (79.03) | 0.02 |
| Invasive hemodynamic monitoring | 175 (4.73) | 177 (4.78) | 0.00 |
| MCS | 181 (4.89) | 174 (4.70) | 0.01 |

SMD, standardized mean difference; CAD, coronary artery disease; MI, myocardial infarction; CVA, cardiovascular accident; PPM, permanent pacemaker; ICD, implantable cardioverter defibrillator; MCS, mechanical circulatory support.
